# Identifying the Salient Genes in Microarray Data: A Novel Game Theoretic Model for the Co-Expression Network

**DOI:** 10.3390/diagnostics10080586

**Published:** 2020-08-13

**Authors:** Papori Neog Bora, Vishwa Jyoti Baruah, Surajit Borkotokey, Loyimee Gogoi, Priyakshi Mahanta, Ankumon Sarmah, Rajnish Kumar, Stefano Moretti

**Affiliations:** 1 Department of Mathematics, Dibrugarh University, Dibrugarh 786004, India; papori2011@gmail.com; 2 Centre for Biotechnology and Bioinformatics, Dibrugarh University, Dibrugarh 786004, India; 3 Department of Applied Mathematics, Northwestern Polytechnical University, Xi’an 710072, China; loyimeegogoi@gmail.com; 4 Centre for Computer Science and Applications, Dibrugarh University, Dibrugarh 786004, India; priyakshi.online@gmail.com (P.M.); ankumonsarmah2009@gmail.com (A.S.); 5 Economics Group, Queen’s Management School, Queen’s University, Belfast BT9 5EE, UK; 6 Université Paris-Dauphine, PSL Research University, CNRS, LAMSADE, 75016 Paris, France; stefano.moretti@dauphine.fr

**Keywords:** co-expression network, colon cancer, cooperative games, microarray, network game, link relevance index, shapely index

## Abstract

Microarray techniques are used to generate a large amount of information on gene expression. This information can be statistically processed and analyzed to identify the genes useful for the diagnosis and prognosis of genetic diseases. Game theoretic tools are applied to analyze the gene expression data. Gene co-expression networks are increasingly used to explore the system-level functionality of genes, where the roles of the genes in building networks in addition to their independent activities are also considered. In this paper, we develop a novel microarray network game by constructing a gene co-expression network and defining a game on this network. The notion of the Link Relevance Index (LRI) for this network game is introduced and characterized. The LRI successfully identifies the relevant cancer biomarkers. It also enables identifying salient genes in the colon cancer dataset. Network games can more accurately describe the interactions among genes as their basic premises are to consider the interactions among players prescribed by a network structure. LRI presents a tool to identify the underlying salient genes involved in cancer or other metabolic syndromes.

## 1. Introduction

The occurrence or activity of the gene product from its coding gene can be investigated through gene expression analyses. The study of gene expression profiling of cells and tissue has become a major tool for discovery in medicine [1]. It is a profound indicator of biological activity where a change in a biological process results from a changing gene expression pattern. Gene expression data analysis requires suitable tools for storing and managing relevant data. Microarrays have been identified as a promising technology to generate huge amounts of information related to the gene expression data [2,3].

DNA microarray experiments permit the portrayal of genome-wide expression variations in various areas like disease and health. These DNA microarrays store data in a consistent expression data matrix. Microarrays have been progressively applied in various medical and biological research activities to solve an array of glitches ranging from human tumor detection to environmental stress alleviation [4,5]. Along with contemporary sequencing tools, the microarray technique continues to be an exceptional methodology for large-scale expression analysis and concepts commonly used in genomic technologies. Microarrays can be utilized for conducting very high-end parallel tests of DNA, RNA, proteins, etc., for expression analysis, the detection of mutation, or re-sequencing [6]. Microarrays have an inherent capacity to spatially sort molecular species such that their concentrations can be autonomously estimated [7].

Genes portraying coordinated expression across a wide range of experimental settings indicate the incidence of functional linkages between genes. Gene co-expression networks can be used for candidate disease gene prioritization, functional gene annotation, and the identification of regulatory genes. Co-expression networks are effectively only able to identify correlations; they indicate which genes are active simultaneously, which often indicates that they are active in the same biological process, but do not normally confer information about causality or distinguish between regulatory and regulated genes [8,9]. Thus, co-expression gene networks can associate the genes of unknown function with biological processes in an intuitive way [10,11]. Co-expression networks are connection situations based on the extent of correlation between pairs of genes across a gene expression dataset. There have been a number of studies that support the flexibility of co-expression analysis for inferring and annotating gene functions [12,13,14,15].

The statistical tests for differential gene expression analysis provide the details of the candidate genes having, individually, a sufficiently low p-value. Nevertheless, it is a challenging task to interpret each single p-value for complex systems that involve numerous interacting genes. Thus, a method for gene expression analysis based on game theory is proposed [16], wherein a class of microarray games is introduced to quantitatively evaluate the relevance of each gene in generating or regulating the condition of the onset of disease. The main advantage of this approach is the possibility to compute a numerical index, i.e., a relevance index, which represents the relevance of each gene under a certain condition taking into account the expression behavior of the other genes under the same condition.

A supplementary feature of this game theoretic approach is that it provides an innovative property-driven classification of the Shapley value in order to contextualize and validate the use of the Shapley value as a significance index for genes. In some studies, several salient genes are identified according to the Shapley value, and their relations with the pathogenesis of neuroblastic tumors are evaluated [17,18]. Microarray games have been used to quantitatively evaluate the relevant genes involved in disease manifestation [16,19,20]. The Shapley value attributed to a certain gene in a given microarray game corresponds to the relevance of that gene for the mechanisms governing the genomic effects of the condition under study. Further, it provides a characterization of a relevance index for genes, which is mainly based on the role they play inside gene-regulatory pathways (GRP). The identification of salient genes that mediate cancer etiology, progression, or therapy response is a challenging task due to the complexity and heterogeneity in cancer biology.

Gene interactions prescribed by some network structure among the participating genes are a recent area of study. The potential applications of network analysis include determining protein or gene function, designing effective strategies for treating various diseases, or providing the early diagnosis of disorders. The information of microarray data must be statistically processed and analyzed to identify the genes that are useful for the diagnosis and prognosis of genetic diseases. Various game theoretic tools are applied to analyze the gene expression data [16,18,21,22,23]. All these techniques have been proposed to identify the genes that have various important roles in the onset of a genetic disease.

In this paper, we develop a model of network games to analyze the microarray data of gene co-expression networks under the cooperative framework. While considering such a network, both genes and their connecting links play an imperative role in shaping its overall structure, and therefore, the Shapley value should be substituted by its network counterpart. The standard values for network games are the Myerson value, which is a player based value or allocation rule, and the position value, which is a link based value or allocation rule [24]. The choice of a particular type of value, player based or link based, depends on the physical problem. If players are more important, we adopt the player based rule, and if the links are more important, we take the link based rule. In our present work, we focus on the gene co-expression networks and the network game over such co-expression networks. Therefore, our emphasis is more towards the linking abilities of the genes that lead to the introduction of the Link Relevance Index (LRI) as a suitable candidate for explaining the relevance of the genes rather than the player based values. We argue that network games can more accurately describe the interactions among genes as they consider not only the cooperation among agents (genes), but also account for how the agents (genes) are connected in a network. We evaluated LRI for the gene co-expression networks, which is analogues to the Shapely value. Therefore, our study involves a more detailed description of genetic markers and their combined effects.

Throughout this paper, we work on a matrix of gene expression values that have been already pre-processed, according to the previous methods. Firstly, we build the theoretical background of the gene co-expression network games, propose the LRI of a network game as a solution representing the significance of each of the genes, and finally, compare the results obtained from the existing methods with our results. The LRI, as we see later, stresses more the links formed by the genes and their respective contributions in the network.

## 2. Materials and Methods

We recall some basic concepts related to the development of our model from [9,16,17,18,21,23,25,26,27,28] in Section 2.1, Section 2.2, Section 2.3. In Section 2.4, we introduce the microarray network games and the corresponding LRI. We also obtain a characterization of the LRI in the context of gene expression networks.

### 2.1. Cooperative Games with Transferable Utilities

Let N={1,2,…,n} be a finite set of players and $2^{N}$ the power set of *N*, i.e., the set of all the subsets of *N*. A cooperative game with Transferable Utilities (TU) is a pair (N,v), where $v:2^{N}\to R$ is the characteristic function with v(∅)=0. Every subset *S* of *N* is called a coalition, and its worth is given by the real number v(S). The set *N* of all the players is called the grand coalition. The class of all TU-games on the player set *N* is denoted by G(N). The main assumption in TU-games is that the grand coalition *N* will eventually form. A solution is a function $\Phi :G\left(N\right)\to R^{n}$ that assigns a vector $\Phi \left(v\right)\in R^{n}$ to each game v∈G(N). The Shapley value, which assigns to each player his/her average marginal contribution over all the coalitions, is perhaps the most popular solution concept that builds on some standard rationality axioms [29]. Formally, given a TU-game (N,v), for each player i∈N, the Shapley value Φ(v) is defined by,

(1) $$\begin{array}{c} \Phi_{i}\left(v\right)=\sum_{i\in S\subseteq N}\frac{(s-1)!(n-s)!}{n!}\left[v\left(S\right)-v\left(S\backslash i\right)\right] \end{array}$$

where s=|S| and n=|N| are the cardinalities of coalitions *S* and *N*, respectively.

An alternative representation of the Shapley value can be given as:

(2) $$\begin{array}{c} \Phi_{i}\left(v\right)=\sum_{i\in S\subseteq N}\frac{\lambda_{S}\left(v\right)}{s}\;\;\mathrm{for}\mathrm{each}\;i\in N, \end{array}$$

where the coefficients ${\left(\lambda_{S}\left(v\right)\right)}_{\left(S\in 2^{N}\right)}$ are called the Harsanyi dividends [30] and given by,

$$\lambda_{S}\left(v\right)=\sum_{T\subseteq S}{\left(-1\right)}^{s-t}v\left(T\right).$$

### 2.2. Microarray Games

Microarray games were defined as TU-games in [16] that account for the relevance of groups of genes in relation to a specific condition. A Microarray Experimental Situation (MES), which is the basis of the microarray games, is defined as follows (see [16] for more details).

Let N={1,2,⋯,n} be a set of *n* genes, $S_{R}=\left\{s_{1}^{R},\ldots ,s_{r}^{R}\right\}$ a set of *r* reference samples, i.e., the set of cells from normal tissues, and $S_{D}=\left\{s_{1}^{D},\ldots ,s_{d}^{D}\right\}$ be the set of cells from tissues with a genetic disease. In a microarray experiment, each sample $j\in S_{R}\cup S_{D}$ is associated with an expression profile $A\left(j\right)={\left(A_{ij}\right)}_{i\in N}$, where $A_{ij}\in R$ represents the expression value of the gene *i* in sample *j*. These expression values are called the dataset of the microarray experiment. The dataset allows for comparison among the expression intensities of genes from different samples. These datasets are presented as two real-valued expression matrices $A^{S_{R}}={\left(A_{ij}^{S_{R}}\right)}_{i\in N;j\in S_{R}}$ and $A^{S_{D}}={\left(A_{ij}^{S_{D}}\right)}_{i\in N;j\in S_{D}}$. An MES is the tuple $E=<N;S_{R};S_{D};A^{S_{R}};A^{S_{D}}>$. In practice, the genes from the samples in $S_{D}$ that are abnormally expressed with respect to the set $S_{R}$ are distinguished according to some discriminant function *m*. The overexpressed genes pertaining to the discriminant function *m* are assigned one and the normal ones zero. Thus, each MES can be represented by a Boolean matrix $B\in {\left\{0,1\right\}}^{n\times k}$, where k≥1 is the number of arrays with the Boolean values (zero and one). A coalition S⊆N that realizes the association between the expression property and the condition on a single array is called a winning coalition for that array. Let $B_{.j}$ be the *j*th column of B. The support of $B_{.j}$, denoted by $sp(B_{.j})$ is the set $sp\left(B_{.j}\right)=\left\{i\in \left\{1,\ldots ,n\right\}\;\;\mathrm{such}\mathrm{that}\;\;B_{ij}=1\right\}$.

The microarray game corresponding to B is the TU-game (N,v), where $v:2^{N}\to R$ is such that v(T) denotes the rate of occurrences of coalition *T* as a winning coalition, i.e., as a superset of the supports in the Boolean matrix B. Formally, for each $T\in 2^{N}\backslash \left\{\emptyset \right\}$, v(T) is the value given by,

(3) $$\begin{array}{c} v\left(T\right)=\frac{\left|\Theta (T)\right|}{|S_{D}|} \end{array}$$

where |Θ(T)| is the cardinality of the set $\Theta \left(T\right)=\{j\in K:sp\left(B_{.j}\right)\subseteq T,sp\left(B_{.j}\right)\neq \emptyset \}$. The class of microarray games is denoted by the symbol M. The Shapley value is shown to be a solution to the microarray games by genetically interpreting its properties.

### 2.3. Network Game

Let N={1,2,…,n} be a nonempty set of players that are connected in some network relationship. A link is an unordered pair of players {i,j}, where i,j∈N. For simplicity, write ij to represent the link {i,j}. The set $g^{N}$ ={ij:i,j∈N,i≠j} of all subsets of *N* of size two is called the complete network. Let $G=\{g:g\subset g^{N}\}$ denote the set of all possible networks on *N*. With an abuse of notation, by ij∈g, we mean that *i* and *j* are linked under the network *g*. For instance, if N={1,2,3}, then g={12,23} is the network where there is a link between Players 1 and 2 and another link between Players 2 and 3, but there is no link between Players 1 and 3. Therefore, with the above notation, we have 12∈{12,23} and similarly 23∈{12,23}. Let N(g) be the set of players who have at least one link in *g*; that is, N(g)={i|∃j∈N;suchthatij∈g}. Let n(g)=|N(g)| denote the number of players involved in *g*. Take |g| to be the number of links in *g*. By $g_{i}$, we denote the set of links that player *i* is involved in *g*, so that $g_{i}=\left\{ij\;|\;\exists \;j\in N\;:\;ij\in g\right\}$. The number of elements in $N(g_{i})$ given by $n(g_{i})$ is also called the degree of the node i∈N in the network *g* and is denoted by deg(*i*). For any $g_{1}$, $g_{2}\in G$, denote by $g_{1}+g_{2}$ the network obtained through adding networks $g_{1}$ and $g_{2}$ and by $g_{1}\backslash g_{2}$ the network obtained from $g_{1}$ by subtracting its subnetwork $g_{2}$. With an abuse of notation, we use g\ij to denote g\{ij} for every link ij∈g. A path in a network g∈G between players *i* and *j* is a sequence of players $i_{1},\ldots ,i_{k}$ such that $i_{k}i_{k+1}\in g$ for each k∈{1,…,n−1}, with $i_{1}=i$ and $i_{k}=j$. The path relationships in a network naturally partition a network into different maximally connected subgraphs that are commonly referred to as components. A component of a network *g* is a non-empty subnetwork $g^{\prime }\subseteq g$ such that:

- if $i\in N(g^{\prime })$ and $j\in N(g^{\prime })$ where j≠i, then there exists a path in $g^{\prime }$ between *i* and *j* and
- if $i\in N(g^{\prime })$ and ij∈g, then $ij\in g^{\prime }$.

Thus, the components of a network are the maximally connected subgraphs of a network. The set of components of *g* is denoted by C(g). Note that $g=⋃g^{\prime }$ for all $g^{\prime }\in C\left(g\right)$. In our framework, we do not consider the isolated players, i.e., the nodes without any link as components.

**Definition** **1.** *A function v:G→R with the condition v(∅)=0 is called a value function where *∅* denotes the empty network. The set of all value functions on G is denoted by V. Under the standard addition and scalar multiplication of functions, namely (v+w)(g)=v(g)+w(g) and (αv)(g)=αv(g) for each v,w∈V and α∈R, V is a linear space.*

**Definition** **2.** *Given g∈G, each of the following special value functions makes a basis for V.*

(4) $$v_{g}\left(g^{\prime }\right)=\left\{\begin{array}{cc} 1\; & \mathrm{if}g\subseteq g^{\prime }\\ 0 & \mathrm{otherwise} \end{array}\right.$$

(5) $${\hat{v}}_{g}\left(g^{\prime }\right)=\left\{\begin{array}{cc} 1\; & \mathrm{if}g{\;}_{\begin{array}{c} \subset \\ \neq \end{array}}\;g^{\prime }\\ 0 & \mathrm{otherwise} \end{array}\right.$$

*and,*

(6) $$v_{g}^{*}\left(g^{\prime }\right)=\left\{\begin{array}{cc} 1\; & \mathrm{if}g=g^{\prime }\\ 0 & \mathrm{otherwise} \end{array}\right.$$

Note that the notion of a basis in *V* is critical to axiomatizing the solution concepts. Since each value function is a linear combination of its basis vectors, the corresponding characterization of a solution in terms of the basis vectors ensures the same characterization of the original game.

**Definition** **3.** *A value function v∈V is component additive if:*

$$v\left(g\right)=\sum_{g^{\prime }\in C\left(g\right)}v\left(g^{\prime }\right),\;\;\mathrm{for}\mathrm{any}\;\;g\in G.$$

**Definition** **4.** *A network game is a pair, (N,v), where N is a set of players and v is a value function on V. If the player set N is fixed, we denote a network game (N,v) simply by the value function v.*

**Definition** **5.** *An allocation rule is a function Y:G× V $\to R^{n}$ that assigns a value $Y_{i}\left(g,v\right)$ to each player i∈N.*

Thus, an allocation rule in a network game describes how the value generated by the network is allocated among the players. For a survey on the alternative allocation rules for network games, we recommend [25,31]. An allocation rule *Y* is link based if there exists a function $\Psi :G\times V\to R^{n(n-1)/2}$ such that:

(7) $$\begin{array}{ccc} \sum_{ij\in g^{N}}\Psi_{ij}\left(g,v\right)=v\left(g\right) & \mathrm{and} & Y_{i}\left(g,v\right)=\sum_{i\neq j}\frac{\Psi_{ij}\left(g,v\right)}{2} \end{array}$$

Thus, a link based allocation rule allocates the total worth of a network to the players in two steps: the value is first allocated among the links treating them as players, and then, it is divided equally between the nodes (players) forming each such link. The position value [25,27,28,32] is one of the popular link based allocation rules that is based on the Shapley value [29] of the links in a network. It is denoted by $Y_{i}^{PV}$ and given by (see [28]),

(8) $$\begin{array}{c} Y_{i}^{PV}\left(g,v\right)=\sum_{i\neq j,ij\in g}\left(\sum_{g^{\prime }\subseteq g}\frac{1}{2}\left(v\left(g^{\prime }\right)-v\left(g^{\prime }\backslash ij\right)\right)\right)\frac{\left(\right|g^{\prime }\left|-1)!(|g|-\right|g^{\prime }\left|\right)!}{|g|!}\;\;\; \end{array}$$

An equivalent form of the position value using the unanimity coefficients $\lambda_{g}\left(v\right)$ due to [28] is given below.

(9) $$\begin{array}{c} Y_{i}^{PV}\left(g,v\right)=\sum_{g^{\prime }\subseteq g,i\in N\left(g^{\prime }\right)}\frac{|g_{i}^{\prime }|}{2|g^{\prime }|}\lambda_{g^{\prime }}\left(v\right) \end{array}$$

Observe that the position value in a network game (N,v) receives half of the Shapley value of each of the links in which the player is involved. In what follows next, we present a recent characterization of the position value due to [28]. As an a priori requirement, we state the following definitions.

**Definition** **6.** *An allocation rule Y defined on G×V is additive if:*

$$Y\left(g,v_{1}+v_{2}\right)=Y\left(g,v_{1}\right)+Y\left(g,v_{2}\right),$$

*for each pair $\;\left(N,v_{1}\right),\;\;\left(N,v_{2}\right)$ of network games with component additive value functions $v_{1}$ and $v_{2}$.*

**Definition** **7.** *For i,j∈N, the link ij∈g is superfluous in the network game (N,v) if:*

$$v\left(g^{\prime }\right)=v\left(g^{\prime }\backslash ij\right),$$

*for each network $g^{\prime }\subseteq g$.*

**Definition** **8.** *An allocation rule Y defined on G×V satisfies the superfluous link property if:*

Y(g,v)=Y(g\ij,v)

*for each network game (N,v) with a component additive value function v and all links ij that are superfluous in (N,v).*

The superfluous link property states that if a link in the network is insignificant in terms of the value the network accrues, the allocation rule also does not consider that link for the computation of the value for each player. This idea is similar to the null-player property of TU-games [25].

**Definition** **9.** *A value function v is link anonymous on g if $v\left(g^{\prime }\right)=v\left(g^{\prime \prime }\right)$$\;\forall \;g^{\prime },g^{\prime \prime }\subseteq g$ such that $|g^{\prime }\left|=\right|g^{\prime \prime }|$.*

Link anonymity states that when all the links in a network are interchangeable for the purpose of determining the values of the subnetworks, the relative allocations of the players in the network are determined by the respective number of links in which each player is involved. This idea is similar to that of the symmetry axiom of the Shapley value for TU-games [25].

**Definition** **10.** *An allocation rule Y on G×V is link anonymous if for every network g∈G and link anonymous value function v∈V on g, there exists an α∈R such that:*

$$Y_{i}\left(g,v\right)=\alpha \left|g_{i}\right|,\;\forall \;i\in N.$$

**Definition** **11.** *An allocation rule Y satisfies efficiency if $\sum_{i\in N}Y_{i}\left(g,v\right)=v\left(g\right)$ for all network games (N,v).*

In [28], the following characterization theorem of the position value is proven. This result is used in a later part of this paper.

**Theorem** **1.** *([28], p. 16) The position value $Y^{PV}$ is the unique allocation rule on the domain of all value functions that satisfies efficiency, additivity, the superfluous link property, and link anonymity.*

### 2.4. Microarray Network Games and the Link Relevance Index

To obtain a microarray network game, we construct a gene co-expression network and then define a value function on this network. Recall from Section 1 that the co-expression networks are connection situations based on the extent of correlation between pairs of genes across a gene expression dataset. Here, nodes are genes and connections are defined by the co-expression of two genes. Often, we consider the Pearson correlation coefficient as the initial measure of gene co-expression [8]. This measure is then transformed into an adjacency matrix, according to different alternative statistical procedures. When the network game is fully described, we obtain this network game. The LRI of the nodes are indicative of the salient genes responsible for the onset of a disease. In the following, we first describe how the gene co-expression network is obtained.

#### 2.4.1. Construction of Gene Co-Expression Networks

We follow a general framework for the construction of gene co-expression networks (for details, see [33]). In such networks, each gene corresponds to a node, and nodes are connected if the corresponding genes are significantly co-expressed across appropriately chosen tissue samples. In reality, it is tricky to define the connections between the nodes in such networks. To correlate the degrees of two nodes *i* and *j*, we use the Pearson Correlation Coefficient (PCC). The PCC (or the *r*-value) between two nodes is defined as the covariance of the two nodes divided by the product of their standard deviations. If *N* is the number of samples and $x_{i}$ and $y_{i}$ the expression values of genes *i* and *j* of the corresponding samples, then the PCC is calculated as follows.

(10) $$\begin{array}{ccc} cor(i,j) & = & \frac{N\sum x_{i}y_{j}-\sum x_{i}\sum y_{i}}{\sqrt{N\sum x_{i}^{2}-{\left(\sum x_{i}\right)}^{2}}\sqrt{N\sum y_{i}^{2}-{\left(\sum y_{i}\right)}^{2}}} \end{array}$$

Consider the MES $E=<N;S_{D};S_{R};A^{S_{D}};A^{S_{R}}>$. Construct a real matrix $R^{\left(E,m\right)}$ using a discriminant function *m* on the entries of $A^{S_{D}}$ and $A^{S_{R}}$. In $R^{\left(E,m\right)}$, zeroes represent the normal genes, and the nonzero entries represent the diseased genes with different expression levels of respective samples, which is unlike the Boolean matrix B of a microarray game. From $R^{\left(E,m\right)}$, we obtain the adjacency matrix for the gene co-expression network based on some biologically motivated criterion (referred to as the scale-free topology criterion). This is done by defining first a similarity measure $s_{ij}$ between each pair of genes *i* and *j*. Denote by $s_{ij}$ the absolute value of the Pearson correlation coefficient, |cor(i,j)|. Note that $s_{ij}\in \left[0,1\right]$. Genes with no correlation are assigned a value near 0.0, while genes that are strongly correlated are assigned a value near 1.0. We denote the similarity matrix by $S=[s_{ij}]$. *S* can be considered to be a weighted network.

To transform the similarity matrix into an adjacency matrix, an adjacency function needs to be defined. The adjacency function is a monotonically increasing function that maps the interval [0,1] into {0,1}. The most widely used adjacency function is the signum function, which involves the threshold parameter τ; see [33]. The signum function is defined as follows,

(11) $$a_{ij}=\mathrm{signum}\left(s_{i,j},\tau \right)=\left\{\begin{array}{cc} 1\; & \mathrm{if}s_{i,j}\geq \tau \\ 0 & \mathrm{if}s_{i,j}\leq \tau \end{array}\right.$$

There are several approaches for choosing the threshold parameter τ. Sometimes, information gets lost due to hard thresholding. For example, if two genes are correlated with coefficient 0.79, they are considered to be disconnected with regard to a hard threshold τ=0.8. The signum adjacency function forms an unweighted network. Thus, the gene co-expression network is represented by the adjacency matrix A =$\left[a_{ij}\right]$, where $a_{ij}$ is one if the connection between two nodes *i* and *j* exists and zero otherwise, so the diagonal elements should be zero. Let us denote by $g^{E}$ the gene co-expression network with respect to the MES $E=<N;S_{D};S_{R};A^{S_{D}};A^{S_{R}}>$.

The following example is a slight modification of Example 1 in [16] (pg 259), which highlights the process of obtaining a gene co-expression network from an MES.

**Example** **1.** *Consider the MES $E=<N;S_{D};S_{R};A^{S_{D}};A^{S_{R}}>$ such that the normal sample $A^{S_{R}}$ and the diseased sample $A^{S_{D}}$ are reported in the following tables, respectively.*

$A^{S_{R}}$
Sample 1Sample 2Sample 3Sample 4Gene 10.40.20.30.6Gene 2−121045Gene 34.83.55.56.3Gene 412141719Gene 53.14.67.25.6


$A^{S_{D}}$
Sample 1Sample 2Sample 3Gene 10.90.40.1Gene 2−111318Gene 32.71.95.6Gene 4102015Gene 52.16.31.6

The dataset of a microarray experiment is presented in terms of the logarithms of the relative gene expression ratios of the target sample with the reference sample. A positive number indicates a higher gene expression in the target sample than in the reference one, whereas a negative number indicates a lower expression in the target sample.

Now, construct a real matrix from the expression matrices by using a discriminant method *m* such that for each i∈N and each $j\in S_{D}$:

(12) $${\left(m\left(A^{S_{D}},A^{S_{R}}\right)\right)}_{ij}=\left\{\begin{array}{cc} 0\; & \mathrm{if}\min_{h}A_{\mathrm{ih}}^{S_{R}}\leq A_{\mathrm{ih}}^{S_{D}}\leq \max_{h}A_{\mathrm{ih}}^{S_{R}}\mathrm{such}\mathrm{that}h=\left\{1,2,3\right\}\\ A_{ij}^{S_{D}} & \mathrm{otherwise} \end{array}\right.$$

The corresponding real matrix is:

$$R^{\left(E,m\right)}=\left[\begin{array}{ccc} 0.0 & 0 & 0.1\\ 0 & 13 & 18\\ 2.7 & 1.9 & 0\\ 10 & 20 & 0\\ 2.1 & 0 & 1.9 \end{array}\right]$$

In this matrix, zero represents the normal genes, and the real numbers represent the diseased genes with different expression levels of the respective samples. The similarity matrix *S* with respect to $R^{\left(E,m\right)}$ is given by:

$$S=\left[s_{ij}\right]=\left[\begin{array}{ccccc} 1.0 & 0.93 & 0.65 & 0.10 & 0.65\\ 0.93 & 1.0 & 0.88 & 0.26 & 0.33\\ 0.65 & 0.88 & 1.0 & 0.68 & 0.10\\ 0.10 & 0.26 & 0.68 & 1.0 & 0.81\\ 0.65 & 0.33 & 0.10 & 0.81 & 1.0 \end{array}\right]$$

Considering soft threshold β=1, it follows that *S* represents a weighted network where all genes are connected to each other with some weights. Choosing the power β, the resulting network displays an approximate scale-free topology. However, one potential drawback of the soft threshold is that the network becomes too complex to track the relationship among the nodes. Therefore, the selection of a suitable threshold that allows the connection weights up to a certain level is a critical step. After applying a threshold, we obtain the resulting matrix as an unweighted network. Let us take τ=0.8 for the sake of illustration. Then, the adjacency matrix corresponding to *S* becomes:

$$A=\left[a_{ij}\right]=\left[\begin{array}{ccccc} 0 & 1 & 0 & 0 & 0\\ 1 & 0 & 1 & 0 & 0\\ 0 & 1 & 0 & 0 & 0\\ 0 & 0 & 0 & 0 & 1\\ 0 & 0 & 0 & 1 & 0 \end{array}\right]$$

Thus, $g^{E}=\left\{12,23,45\right\}$ is the required gene co-expression network over the microarray experiment situation *E* and $N\left(g^{E}\right)=\left\{1,2,3,4,5\right\}$. Similarly, for τ=0.6, the corresponding network will be $g^{E}$= $\left\{12,13,15,23,34,45\right\}$.

#### 2.4.2. Microarray Network Games

Once the co-expression network $g^{E}$ has been constructed, i.e., the adjacency matrix has been formed, we have to define a value function *v* on *G*, the set of all possible networks on *N*. Let $N(g^{E})$ and $n(g^{E})$ denote, respectively, the set of genes and the number of genes that form the network $g^{E}$. For instance, in Example 1, $N\left(g^{E}\right)=\left\{1,2,3,4,5\right\}$ and $n(g^{E})=5$.

**Definition** **12.** *Given the co-expression network $g^{E}\in G$, let the support sp(i) of gene i∈N in $g^{E}$ be defined as the set of links in $g^{E}$ that gene i is involved in, i.e., $sp\left(i\right)=\{ij:ij\in g^{E}$ for $j\in N(g^{E})\}$. Therefore, following the standard notations, we have $sp\left(i\right)=g_{i}^{E}$.*

Consider the network $g^{E}=\left\{12,23,45\right\}$ in Example 1. The supports of the respective genes are $sp\left(1\right)=g_{1}^{E}=\left\{12\right\}$, $sp\left(2\right)=g_{2}^{E}=\left\{12,23\right\}$, $sp\left(3\right)=g_{3}^{E}=\left\{23\right\}$, $sp\left(4\right)=g_{4}^{E}=\left\{45\right\}$, and $sp\left(5\right)=g_{5}^{E}=\left\{45\right\}$.

**Definition** **13.** *Let N={1,2,…,n} be the set of genes. Given an MES $E=<N;S_{D};S_{R};A^{S_{D}};A^{S_{R}}>$ and the corresponding gene co-expression network $g^{E}$, a microarray network game with respect to E and $g^{E}$ is the triple $\left(N,v,g^{E}\right)$ where (N,v) is a network game with the value function v that assigns to each g∈G the average number of genes having connections in $g^{E}$. Formally, we define the value function v:G→R as:*

(13) $$\begin{array}{c} v\left(g\right)=\frac{|\hat{C}\left(g\right)|}{n(g^{E})} \end{array}$$

*where $\hat{C}\left(g\right)$ = $\left\{i\in N\left(g^{E}\right):\emptyset \neq g_{i}^{E}\subseteq g\right\}$ for each g∈G.*

Thus, the value function *v* determines the collective influence of a set of genes who are connected through a co-expression network. In practice, v(g) is the average number of genes added over all components that are contained in the set of links where both the genes are involved together in the onset of the disease determined by the network *g*. It follows that an equivalent form of the value function *v* as a sum of the basis games $v_{g}$ defined in Equation (4) in a microarray network game $\left(N,v,g^{E}\right)$ is given by:

(14) $$v=\sum_{\emptyset \neq g\subseteq g^{N}}\alpha_{g}\left(v\right)v_{g}=\sum_{\emptyset \neq g\subseteq g^{N}}\frac{{\bar{\alpha }}_{g}\left(v\right)}{n(g^{E})}v_{g}=\frac{1}{n(g^{E})}\sum_{i\in N\left(g^{E}\right),g_{i}^{E}\neq \emptyset }v_{g_{i}^{E}}$$

where we choose the coefficients $\alpha_{g}\left(v\right)=\frac{{\bar{\alpha }}_{g}\left(v\right)}{n(g^{E})}$ such that ${\bar{\alpha }}_{g}\left(v\right)=\left|\left\{i\in N\left(g^{E}\right):g_{i}^{E}=g\right\}\right|$. If no ambiguity on *N* arises, we denote a microarray network game by the pair $\left(v,g^{E}\right)$. The class of microarray network games with player set *N* is denoted by $M^{N}$.

**Example** **2.** *In Example 1, recall that $g^{E}=\left\{12,23,45\right\}$ is the gene co-expression network and N={1,2,3,4,5} the set of genes. The value function v of the microarray network game $\left(v,g^{E}\right)$ is given by,*

(15) $$v\left(g\right)=\frac{1}{5}\left\{u_{\left\{12\right\}}\left(g\right)+u_{\left\{12,23\right\}}\left(g\right)+u_{\left\{23\right\}}\left(g\right)+u_{\left\{45\right\}}\left(g\right)+u_{\left\{45\right\}}\left(g\right)\right\}$$

*Thus we have, $v\left(\left\{12\right\}\right)=v\left(\left\{23\right\}\right)=\frac{1}{5}$, v({45}) =$\frac{2}{5}$, v({12, 23}) = v({12, 45}) =v({23, 45})= $\frac{3}{5}$, $v\left(g^{E}\right)=v\left(\left\{12,23,45\right\}\right)=1$, and v(g)=0 for all g∈G.*

The value function *v* of the microarray network game $\left(v,g^{E}\right)$ picks up the information that can be used to define the role of each link in each co-expression of genes by applying suitable solution concepts of network games. The value function *v* specifies the total value that is generated by a given network structure. The calculation of the value may involve both costs and benefits in networks and is a richer object than a characteristic function of the microarray game. This is because the value depends on the network structure in addition to the coalition of players involved [26].

#### 2.4.3. LRI for Microarray Network Games and Its Characterization

In the previous subsection, we discussed the allocation rules for network games. An allocation rule for microarray network games describes how the value generated by a network is allocated among the genes. We call it the LRI. Define the function $F:G\times M^{N}\to R^{n}$ on the class of microarray network games as follows.

(16) $$\begin{array}{c} F_{i}\left(g,v,g^{E}\right)=\sum_{g^{\prime }\subseteq g,i\in N\left(g^{\prime }\right)}\frac{|g_{i}^{\prime }|}{2|g^{\prime }|}\alpha_{g^{\prime }}\left(v\right)\\ =\frac{1}{n(g^{E})}\sum_{g^{\prime }\subseteq g,i\in N\left(g^{\prime }\right)}\frac{|g_{i}^{\prime }|}{2|g^{\prime }|}{\bar{\alpha }}_{g^{\prime }}\left(v\right) \end{array}$$

where $\alpha_{g^{\prime }}\left(v\right)$ and, hence, ${\bar{\alpha }}_{g^{\prime }}\left(v\right)$ are defined as in Equation (14). The following example shows the relevance of *F* in Example 2.

**Example** **3.** *Let us consider the network $g^{E}=\left\{12,23,45\right\}$ in Example 2. Using Equation (16), we compute F for different g as follows:*

$$\mathrm{For}g=g^{E}=\left\{12,23,45\right\},\;\;\;F\left(g,v,g^{E}\right)=\left(\frac{3}{20},\frac{6}{20},\frac{3}{20},\frac{4}{20},\frac{4}{20}\right).$$

$$\mathrm{For}g=\{12\},\;\;\;F\left(g,v,g^{E}\right)=\left(\frac{1}{10},\frac{1}{10},0,0,0\right).$$

$$\mathrm{For}g=\{12,23\},\;\;\;F\left(g,v,g^{E}\right)=\left(\frac{3}{20},\frac{6}{20},\frac{3}{20},0,0\right).$$

*The numerical values are indicative of the individual contributions of the genes in the network g, given the microarray network game $\left(v,g^{E}\right)$.*

In what follows next, we define the LRI based on properties similar to the ones that are used to characterize the position value. Recall that the superfluous link property states that the presence or absence of a link between players that has no influence on the value of any network also has no influence on the allocations of respective players in a network. The interpretation of the superfluous link property in the genetic context is simple and intuitive. If a link is deleted from the gene co-expression network, i.e., the expression of two genes along this link is controlled, then the corresponding allocation rule also does not consider the effects of their link. Thus, a link ij∈g is superfluous in the microarray network game $\left(v,g^{E}\right)$ if v(g)=v(g\ij) for all networks g∈G.

**Definition** **14.** *An allocation rule Y on $G\times M^{N}$ satisfies the superfluous gene link property if $Y\left(g,v,g^{E}\right)=Y\left(g\backslash ij,v,g^{E}\right)$ for all microarray network games $\left(v,g^{E}\right)\in M^{N}$ and all links ij that are superfluous in $\left(v,g^{E}\right)$.*

**Proposition** **1.** *F given by Equation (16) satisfies the superfluous gene link property.*

**Proof.**  The proof follows from the simple fact that in a microarray network game $\left(v,g^{E}\right)\in M^{N}$, the superfluous links are those links that are not in $g^{E}$. □

Recall that link anonymity states that when all the links in a network are interchangeable for the purpose of determining the values of the sub-networks, the relative allocations of the players in the network are determined by the relative number of links in which each player is involved. In the context of gene co-expression networks, the anonymity property says that the value of a gene co-expression network is derived from the structure of the network and not the labels of the genes who occupy various positions. Owing to this property, genes survive, swapping from one organism to the other, as recently observed in [34].

**Definition** **15.** *Let the microarray network game $\left(v,g^{E}\right)\in M^{N}$ be link anonymous, i.e., $v\left(g^{\prime }\right)=v\left(g^{\prime \prime }\right)$ for every pair of $g^{\prime },g^{\prime \prime }\subseteq g$ such that $|g^{\prime }\left|=\right|g^{\prime \prime }|$. An allocation rule Y on $G\times M^{N}$ satisfies the gene link anonymity if there exists $\alpha_{i}\in R$ for each i∈N such that $Y_{i}\left(g,v,g^{E}\right)=\alpha_{i}\left|g_{i}\right|$ for each link anonymous microarray network game $\left(v,g^{E}\right)\in M^{N}$.*

**Proposition** **2.** *F given by Equation (16) satisfies link anonymity.*

**Proof.**  Since *F* is a function of the respective sizes of the networks *g*, $g^{E}$, and $g_{j}^{E}$ ($j\in N(g^{E})$), the result follows immediately from the definition. □

Now, we define the LRI for the class of microarray network games as follows.

**Definition** **16.** *An allocation rule $Y:G\times M^{N}\to R^{n}$ is called an LRI on the class of microarray network games if it satisfies efficiency, additivity, the superfluous gene link property, and the gene link anonymity.*

The following is a characterization theorem of the LRI.

**Theorem** **2.** *F given by Equation (16) is the unique LRI on $G\times M^{N}$.*

**Proof.**  The additivity of *F* easily follows from the well-known fact that the unanimity coefficients are additive in value functions. Using Proposition 1 and a result in graph theory that states that $\left|g\right|=\frac{1}{2}\sum_{i\in N}\left|g_{i}\right|$, we have,

$$\begin{array}{cc} \sum_{i\in N}F_{i}\left(g,v,g^{E}\right) & =\sum_{i\in N}\frac{1}{n(g^{E})}\sum_{g^{\prime }\subseteq g}\frac{|g_{i}^{\prime }|}{2|g^{\prime }|}{\bar{\alpha }}_{g^{\prime }}\left(v\right)\\ \; & =\sum_{g^{\prime }\subseteq g}\alpha_{g^{\prime }}\left(v\right)\sum_{i\in N}\frac{|g_{i}^{\prime }|}{2|g^{\prime }|}\\ \; & =\sum_{g^{\prime }\subseteq g}\alpha_{g^{\prime }}\left(v\right)\\ \; & =v(g) \end{array}$$

Thus, we see that *F* satisfies all the axioms of an LRI. For the converse part, let the function $Y:G\times M^{N}\to R^{n}$ satisfy these properties. Then, *Y* can be extended to a function $\tilde{Y}:G\times V\to R^{n}$ that also satisfies these properties. It is straight forward to show that $\tilde{Y}$ is the position value on G×V such that $\tilde{Y}{|}_{G\times M^{N}}=F$. Thus, by the uniqueness of the position value, Y=F. This completes the proof. □

**Remark** **1.** *In particular, when $g=g^{E}$ in Equation (16), an equivalent form of the LRI $F_{i}\left(g^{E},v,g^{E}\right)$ can be obtained as follows. Take $N_{i}\left(g^{E}\right)=N\left(g_{i}^{E}\right)\backslash \left\{i\right\}$ and $n_{j}\left(g^{E}\right)=n\left(g_{j}^{E}\right)-1$. Thus, $N_{i}\left(g^{E}\right)$ denotes the set of neighbors of i in $g^{E}$ (i.e., all the nodes j≠i that are directly connected to i) and $n_{j}\left(g^{E}\right)$ the number of neighbors of node j (that is the degree of j in the graph). Next, consider the game $v_{g_{i}^{E}}$ (refer to Equation (5)) with $g_{i}^{E}\neq \emptyset$. By Theorem 2, $F(g^{E},v_{g_{i}^{E}},g^{E})$ satisfies gene link anonymity. Therefore, we have:*

$$F_{k}\left(g^{E},v_{g_{i}^{E}},g^{E}\right)=\left\{\begin{array}{cc} \frac{1}{2}\frac{1}{|g_{i}^{E}|}, & if\;k\in N(g_{i}^{E})\\ 0, & otherwise. \end{array}\right.$$

*Moreover, by Equation (14) and the additivity of F, we have that:*

$$F\left(g^{E},v,g^{E}\right)=\frac{1}{n(g^{E})}\sum_{i\in N\left(g^{E}\right),g_{i}^{E}\neq \emptyset }F\left(g^{E},v_{g_{i}^{E}},g^{E}\right),$$

*Observe that if $g_{i}^{E}\neq \emptyset$, $F_{i}\left(g^{E},v_{g_{i}^{E}},g^{E}\right)=\sum_{ij\in g_{i}^{E}}\frac{1}{2n(g^{E})}\frac{1}{|g_{i}^{E}|}=\left|g_{i}^{E}\right|\frac{1}{2n(g^{E})}\frac{1}{|g_{i}^{E}|}=\frac{1}{2n(g^{E})}$, while $F_{i}\left(g^{E},v_{g_{j}^{E}},g^{E}\right)=\frac{1}{2n(g^{E})}\frac{1}{|g_{j}^{E}|}=\frac{1}{2n(g^{E})}\frac{1}{n_{j}\left(g^{E}\right)}$, for all $j\in N_{i}\left(g_{i}^{E}\right)$. It follows that,*

(17) $$F_{i}\left(g^{E},v,g^{E}\right)=\frac{1}{2n(g^{E})}\left(1+\sum_{j\in N_{i}\left(g^{E}\right)}\frac{1}{n_{j}\left(g^{E}\right)}\right)\;\;\mathrm{for}\mathrm{all}i\in N.$$

*Equation (17) suggests that, according to the LRI, a node is more important if connected to too many nodes that are not very well connected. This formula is very close (at least in the interpretation) to the Shapley values given in [19,20] for TU-games defined on a gene network. However, the two approaches are completely different both in the game formulation and in the definition of the index. Another important difference between them is that in Equation (17), each node contributes to its relevance a fixed amount of one, whereas in the formula of the Shapley value in [19,20], it contributes with the value of $\frac{1}{n(g_{i}^{E})+1}$.*

## 3. Results and Discussions

We tested our model on a previously reported colon cancer dataset [4,16,35,36] (http://genomics-pubs.princeton.edu/oncology/affydata/index.html.) containing the expression of 2000 genes with highest minimal intensity across 62 tissues. In the expression data measured using Affymetrix oligonucleotide microarrays, forty tumor samples and a set of 22 normal samples exist. An adjacency matrix is obtained using the signum function based hard thresholding approach, which encodes edge information for each pair of nodes in the network. A pair of genes is said to be connected by an edge if their similarity value, which is calculated using the Pearson correlation, is greater than a threshold. We considered the threshold value to be 0.9 for our experiment.

**Figure 1:**
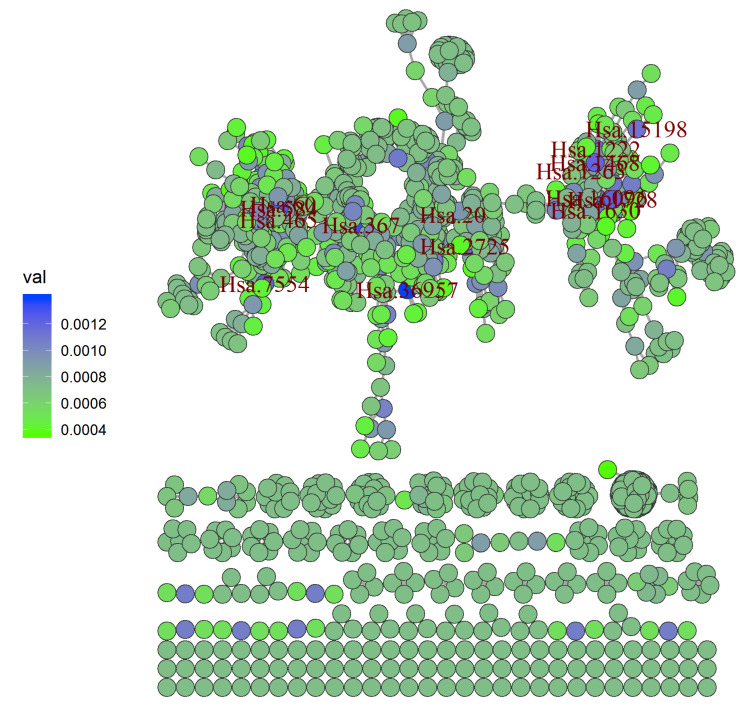
The graph represents the genes in colour based on LRI value. Affy-id of top fifteen genes in colon cancer dataset exhibiting highest LRI values are also labelled.

A network (Figure 1) was constructed employing the LRI on the colon cancer dataset (refer Section 3). The network was made utilizing the igraph [37] package in R [38] by using the adjacency matrix generated after removing isolated points. The colors of the nodes connote the link relevance index varying from least (green) to highest (blue). Affy IDs of the top 15 genes are used to label the highest LRI nodes. The top fifteen genes selected by their highest LRI and its corresponding Shapley values reflect various cellular mechanisms (Table 1). Most of them were previously observed to be associated with the colon cancer.

**Figure 2:**
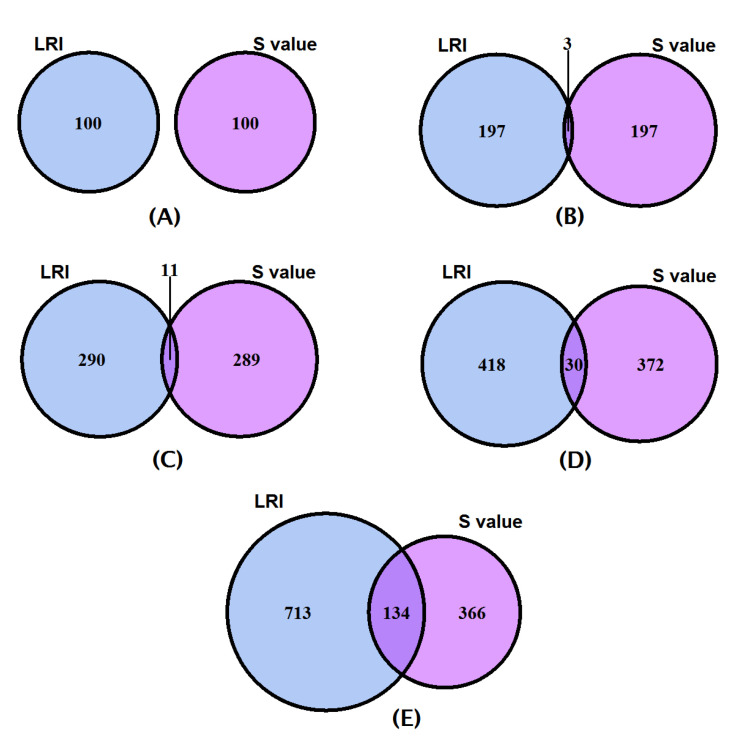
Venn diagrams exhibiting overlaps between the top 100 (**A**), 200 (**B**), 300 (**C**), 400 (**D**), and 500 (**E**) ranked gene sets identified by the LRI (cornflower blue) and Shapley (S) (dark orchid) methods.

We further analyzed if the genes were similarly ranked by the two methodologies viz., the LRI and the Shapely value. The LRI and the Shapely value depict no overlap between the top 100 genes (Figure 2A). However, the top 200, 300, 400, and 500 genes (Figure 2B–E) exhibit 3, 11, 30, and 134 gene overlaps, respectively, between the two indices, suggesting there is a difference in the relative scoring of the genes using the two methodologies and therefore less similarity in the top selected gene sets.

**Figure 3:**
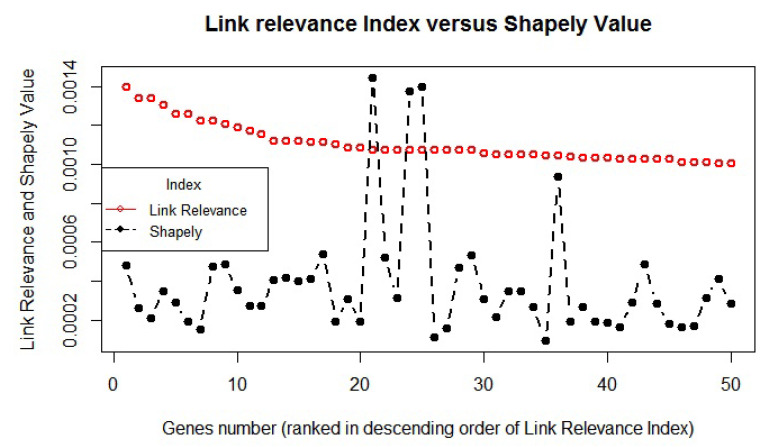
The relationship between the LRI and its corresponding Shapely value of the genes. The LRI (red hollow circle) of the top 50 genes and corresponding Shapely value (black solid circle) are plotted to analyze any similarity between them.

The LRI and the corresponding Shapely value of top 50 genes are plotted to analyze any link/similarity between them (Figure 3). We found that the distribution of the LRI score of the top genes was not only different than the Shapely value, but also their distribution may follow a varied trend due to the likely difference in the background ranking method. Furthermore, Pearson’s correlation also suggests no significant correlation ($R^{2}=0.0833$) between the LRI and Shapely value. The two methods were found to be separate in terms of their overall findings, and therefore, the LRI was considered to be a unique approach rather than a derived one.

**Figure 4:**
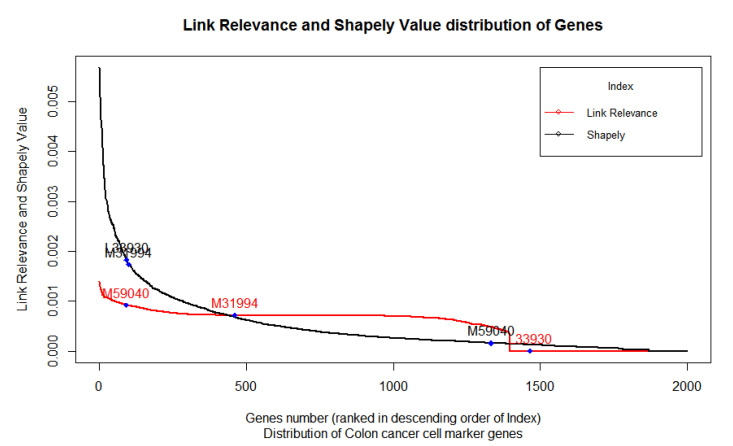
The LRI and Shapley Value score of gene sets arranged in a rank-wise manner and the position of the three biomarkers relative to each other.

We retrieved the list of all marker genes from the CellMarker database [16,39] that were well characterized and validated through the experimental setup and not just through theoretical estimation. Thereafter, we mined these marker genes to corresponding gene names and mapped them against the probe in the microarray platform. Three IDs viz. “Hsa.1240”, “Hsa.654”, and “Hsa.663“ corresponding to genes ALDH1A1(M31994), CD24 (L33930), and CD44(M59040), respectively, were selected for further analysis, as can be seen in Figure 4.

Figure 4 exhibits the distribution of the LRI of 2000 genes from highest to lowest in a rank-wise manner for each gene. We also plot the position of the three biomarkers, namely (CD44) M59040, (ALDH1A1) M31994, and (CD24) L33930, to show their relative position in this distribution. Shapely values of corresponding microarray games, arranged from highest to lowest, are also presented to compare the distribution pattern and relative position of the three biomarkers. LRI was able to correctly estimate the expected relative position of these colon cancer biomarkers. On the one hand, the Shapely value exhibited an exponential increase in the score, the LRI, which is based on the contribution of each gene in the co-expression network, exhibited a nonlinear curve in the distribution of the scores of 2000 genes.

Colon Cancer Stem Cells (CCSCs) not only have the potential of self-renewal and differentiation, but also exhibit “tumorigenicity” when transplanted into an animal host. CD44 (M59040) expressed on the surface of the CCSC is reported to have a major role in the progression, survivability, and “tumorigenicity” of such CCSCs, thereby making it a potent biomarker and target for diagnosis, biosensing, prognosis, and therapeutics in the case of colon cancer [40,41,42,43]. Du L et al. (2008) [41] reported the relevance of CD44 as a superior marker and its functional significance in contributing to CCSCs for cancer initiation and progression.

We found the LRI was able to estimate the higher relevance of CD44 (M59040) by means of estimating its contribution in the co-expression network by assigning it higher index of relevance. On the other hand, the same gene scored poorly in the Shapely value, which undermines its relevance. This validates that the LRI is better able to estimate the relevance of the gene compared to the Shapely value (Table 2, Figure 4).

The gene M31994 encodes Aldehyde dehydrogenase 1A1 (ALDH1A1), which catalyzes aldehydes to their corresponding carboxylic acids through the oxidation process [44]. It has also been enunciated that a considerable amount of ALDH1A1 enrichment occurs in colon cancer [45,46]. ALDH1A1 has been successfully used as a CCSC marker along with many other cancers, including breast cancer [47,48]. However, studies evaluating the association/relationship between ALDH1A1 expression with colon cancer initiation and progression for prognosis and therapeutics remain inconclusive [49,50,51,52,53]. Scientists have argued about the significance of the role of ALDH1A1 in colorectal cancer. Furthermore, clinical evidence equivocally suggests ALDH1A1’s application as a prognostic or predictive biomarker in colon cancer [50]. Moreover, most of the aforementioned research articles did mention the role of CD44 along with ALDH1A1 in cancer initiation, progression, and metastasis.

The gene M31994’s (ALDH1A1) relevance in the control case dataset of colon cancer was found to be moderate using the LRI. However, for the Shapely value, the same gene scored very high along with L33930 (CD24). The LRI method was better able to estimate its position relative to M59040 (CD44) compared to the Shapely value.

CD24 is the product of the L33930 gene and is anchored on the exterior side of the cell membrane. The positive expression and overabundant distribution of CD24 in colorectal cancer is under dispute [52]. A few previous studies reported that CD24 was expressed higher in a fraction of the colorectal cancer population [54,55]. Furthermore, researchers asserted CD24 expression to be limited to only a small fraction of colon cancer cell lines [56]. However, none of these previous reports refuted the significant role of CD44 in colon cancer cell lines. Instead, experimental evidence indicated that CD44 expression was highly significant in the considered colon cancer cell lines, thus highlighting its importance in colon cancer development and progression, but maintaining that only a fraction of these cells exhibited the expression of CD24 [52,54,55,56]; in the authors own words, at “a fair level of 5–10%” [56]. They reported that HCT116 and SW480 colon cancer cells were CD44+ cells and that only a subpopulation of these CD44+ cells exhibited CD24 [56]. Evidence based on clinical studies not only highlighted the marginal contribution of CD24 [52,56], but also stressed CD44 expression in CCSC in initiating cancer, thus making it a better biomarker for colon cancer [41,52].

While comparing the three biomarkers, LRI rightfully estimated the marginal contribution of L33930 (CD24) in colon cancer development and progression; however, the Shapely value scored it very high compared to M59040 (CD44). The Shapely values scored L33930 (CD24) highest among all three genes, despite the previous experimental evidences suggesting its relatively lower relevance. The LRI, however, was able to predict the relative relevance of this gene and positioned it after M59040 (and M31994). In fact, the LRI was able to predict that L33930’s (CD24) role is only incidental and that its expression has no or marginal contribution to colon cancer.

Compared to the Shapley value, the LRI was able to identify the relative contribution/position of the three colon cancer biomarkers. The relevance of same three biomarkers is also evident from experimental studies, including high-throughput single cell RNA seq, as mentioned in the PanglaoDB [57].

### Pseudocodes for the Gene Co-Expressions Networks’ Formation

The symbols given in Table 3 are useful in describing our method. The pseudocodes of the proposed method is presented in Algorithm 1.

**Algorithm 1:** Pseudocode of the gene co-expression network’s construction.**INPUT:** D and τ
**OUTPUT:**
$g^{E}$

**for**$g_{i}\in$, **do**Compare $A^{SD}$ with $A^{SR}$
**end for**
*R* is constructed after comparing**for** For i=1 to *n*, **do***S* is constructed using Pearson correlation
**end for**
**for** For i=1 to m, **do**A is obtained by taking τ=0.9
**end for**
$g^{E}$ is obtained from A


## 4. Conclusions

The identification of salient genes that mediate cancer etiology, progression, or therapy response is a challenging task due to the complexity and heterogeneity in cancer data. In a network game, the challenge is to find how players form a network, accrue a value due to the formation of the network, and finally, allocate the value of the network among the participating players. In this paper, we introduced the notion of a microarray network game to highlight the application of network games in gene expression analysis related to disease onset. We obtained the Link Relevance Index (LRI) to highlight the significance of the genes in a Microarray Experimental Situation (MES). By analyzing a real-world dataset, we made a comparison of our model with the existing game theoretic model in identifying the salient genes responsible for colon cancer. Indexing of genes according to the Shapely values rarely identified genes according to the expectation. The LRI model was validated by its ability to identify the relative relevance of three biomarkers of colon cancer. The results of the analysis on these biomarkers established not just the validity of the Link Relevance (LR) method, but also its advantage compared to the Shapely value in its ability to find the salient genes. In all three biomarker cases, the LR was able to score the genes according to their relative relevance and thus was able to identify salient genes in comparative expression studies. Moreover, in comparison to the Shapely value, the results of the LR method are close to actual immuno-histo-chemical assays and cancer genetic experiments reported previously. These results suggest that our proposed model is superior, and the top genes in the network show their contribution towards the development of colon cancer. The proposed model can be extended to study similar problems related to other genetic or metabolic syndromes.

## Figures and Tables

**Table 1:** Top 15 genes with highest LRI and its corresponding Shapley value.

SlNo.	Gene No.	Annotation	LRI	Shapley Value
1	H43908	Transforming growth factor beta 2 precursor (Gallus gallus)	0.001398	0.000482
2	D17400	Human mRNA for 6-pyruvoyl-tetrahydropterin synthase	0.00134	0.000264
3	D12686	Human mRNA for eukaryotic initiation factor 4 gamma (eIF-4 gamma)	0.001339	0.000212
4	D00762	Proteasome component C8 (human)	0.001303	0.00035
5	U07695	Human Tyrosine Kinase (HTK) mRNA	0.001261	0.00029
6	J03569	Human lymphocyte activation antigen 4F2 large subunit mRNA	0.001261	0.00019
7	R46069	Merozoite surface antigens precursor (Plasmodium falciparum)	0.001225	0.000152
8	X17097	Human PSG9 mRNA for pregnancy specific glycoprotein 9.	0.001223	0.000476
9	M31679	Human Gastric Inhibitory Polypeptide (GIP) gene, exon 6.	0.001208	0.000484
10	H07899	Vascular endothelial growth factor precursor (Homo sapiens)	0.001188	0.000353
11	M19283	Human cytoskeletal gamma-actin gene	0.001174	0.000274
12	X14767	Human mRNA for GABA-A receptor, beta 1 subunit	0.001157	0.000271
13	M97370	Human Adenosine receptor (A2) gene	0.001122	0.000408
14	M69238	Human Aryl hydrocarbon Receptor Nuclear Translocator (ARNT) mRNA	0.001121	0.000417
15	T77537	Plasminogen (Sus scrofa)	0.00112	0.000397

**Table 2:** List of well-validated biomarkers for colon cancer stem cells from the validated experimental setup (along with a review for more reference and importance) retrieved from the CellMarker database [39] and populated thereafter (for the ease of reading).

Tissue	Cell Type	Cell Marker				Source	Details (PubMed ID)
		CD44	CD24	ALDH1A1	Others		
		*	*		*	Experiment	26399781, 29277789
		*			*	Experiment	25625240, 22310487
		*		*	*	Experiment	26185996
	Cancer	*		*	*	Experiment	21196254
Colon	stem	*			*	Experiment	27806848
	cell	*	*		*	Experiment	27789195
		*				Experiment	27323782
		*	*		*	Experiment	28986882
		*			*	Review	22459349

Asterisk in table means a yes mark.

**Table 3:** Symbol table.

Symbols	Term
*D*	The gene expression dataset
$A^{SD}$	Disease dataset
$A^{SR}$	Normal dataset
*R*	Real matrix
*S*	Similarity matrix
$g^{E}$	Link matrix
τ	Similarity threshold
$g_{i}$	ith gene in D

## Abbreviations

The following abbreviations are used in this manuscript:

ALDH1A1	Aldehyde dehydrogenase 1A1
CD	Cluster of Differentiation
CCSCs	Colon Cancer Stem Cells
MES	Microarray Experimental Situation
LRI	Link Relevance Index
